# Glucocorticoid Insensitivity in Asthma: The Unique Role for Airway Smooth Muscle Cells

**DOI:** 10.3390/ijms23168966

**Published:** 2022-08-11

**Authors:** Patricia Ramos-Ramírez, Omar Tliba

**Affiliations:** Joint Health Science Center, School of Veterinary Medicine, Rowan University, 201 Broadway, Camden, NJ 08103, USA

**Keywords:** airway remodeling, airway inflammation, asthma, glucocorticoids, corticosteroids, steroid resistance, airway smooth muscle, glucocorticoid receptor beta, kinases, phosphatases

## Abstract

Although most patients with asthma symptoms are well controlled by inhaled glucocorticoids (GCs), a subgroup of patients suffering from severe asthma respond poorly to GC therapy. Such GC insensitivity (GCI) represents a profound challenge in managing patients with asthma. Even though GCI in patients with severe asthma has been investigated by several groups using immune cells (peripheral blood mononuclear cells and alveolar macrophages), uncertainty exists regarding the underlying molecular mechanisms in non-immune cells, such as airway smooth cells (ASM) cells. In asthma, ASM cells are among the targets of GC therapy and have emerged as key contributors not only to bronchoconstriction but also to airway inflammation and remodeling, as implied by experimental and clinical evidence. We here summarize the current understanding of the actions/signaling of GCs in asthma, and specifically, GC receptor (GR) “site-specific phosphorylation” and its role in regulating GC actions. We also review some common pitfalls associated with studies investigating GCI and the inflammatory mediators linked to asthma severity. Finally, we discuss and contrast potential molecular mechanisms underlying the impairment of GC actions in immune cells versus non-immune cells such as ASM cells.

## 1. Introduction

Asthma is a complex chronic airway disease characterized by reversible airway obstruction, airway hyperresponsiveness (AHR), airway inflammation, and airway structural changes [1]. Glucocorticoids (GCs) are considered the most effective anti-inflammatory agents used to treat patients with inflammatory lung disease [2]. However, prolonged treatment with GCs, particularly when used at high doses, may result in a variety of side effects such as adrenal suppression, diabetes, glaucoma, myopathy, and osteoporosis, therefore limiting the therapeutic potential of their systemic use [3,4,5,6]. Furthermore, despite the effectiveness of GCs in most patients with asthma, a significant subpopulation (5–10%) remains insensitive to GCs [7]. Asthma in individuals insensitive to GCs is often difficult to manage and exhibits the severe form of the disease. In fact, the reduction in GC sensitivity in patients with asthma appears to correlate with disease severity [8].

Undoubtedly, airway smooth muscle (ASM) plays a key role in airway dysfunction, mainly due to the increased thickness of the ASM layer seen in patients with severe asthma [9,10,11,12]. Thickening of the ASM layer is a pivotal component of airway remodeling that underpins exaggerated airway narrowing in asthma [13]. Interestingly, ASM contributes to a plethora of processes associated with disease progression through its different phenotypes. For instance, ASM cells derived from patients with asthma exhibit increased proliferative [14,15], contractile [16], and/or synthetic/secretory responses [1,17,18,19] when compared to ASM cells derived from patients without asthma [16]. Several studies showed that ASM cells secrete a variety of inflammatory mediators (reviewed elsewhere [17]) such as growth factors (TGFβ, IGF, CTGF, VEGF, PDGF-BB), extracellular matrix proteins (collagen, laminin, perlecan), type 1 (T1) and type 2 (T2) cytokines, peptides (bradykinin), and chemokines (eotaxin, RANTES, CXCL8). These reports clearly indicate that ASM actively contributes to the development and perpetuation of airway inflammation [1,17,18,19,20,21,22]. These reports also propose ASM as an important target for anti-inflammatories such as GCs and may contribute to the lack of GC sensitivity seen in patients with severe asthma. Future efforts aimed at developing therapeutic strategies that improve the effectiveness of GC therapy and/or restore GC responsiveness in patients with severe asthma should take into consideration the role that could be played by ASM cells.

In this review, we discuss our current understanding of the actions/signaling of GCs in asthma, the “common pitfalls” associated with studies investigating GCs and GC insensitivity, and the inflammatory mediators linked to asthma severity and GC insensitivity in airway structural cells. We also introduce ASM as a likely contributor to GC insensitivity seen in patients with severe asthma by discussing and contrasting examples of potential molecular mechanisms underlying the impairment of GC actions in immune cells versus ASM cells.

## 2. Glucocorticoid Signaling

### 2.1. The Human GC Receptor (hGR)

The hGR is a 777-amino-acid-long transcription factor encoded by the Nr3cl gene (nuclear receptor 3, group C, member 1) on the long arm of chromosome 5 (5q31Y32) [23,24]. This gene comprises nine exons, where splicing of exon 1 gives rise to at least five different 5′ untranslated regions (UTR) in the human GR: exon 1A1, 1A2, 1A3, 1B, or 1C [25]. These regions may affect post-transcriptional gene events such as the stability, the export, and the processing of mRNA [26]. Exons 2–9 embody the coding region of the GR gene and can undergo alternative splicing processes, thereby generating several GR isoforms, i.e., GRα, GRβ, GRγ, GR-A, and GR-P. To date, the study of GR biology has mainly focused on GRα and GRβ isoforms, which are derived from alternative splicing of exon 9 [27]. GRα is the predominant isoform in airway cells [28]. Although being 94% identical, the GRβ isoform has a divergent carboxyl terminal in the ligand-binding domain, preventing it from binding to GCs. As such, GRβ interferes with GRα function, where it acts as a dominant negative receptor [29]. Higher than normal levels of the GRβ isoform have been observed in GC-resistant patients [29,30]. To date, GRβ has been described to be expressed in immune cells, particularly in neutrophils [23]. The expression and function of GRβ in airway structural cells and ASM cells, in particular, and its possible implications in GC insensitivity in asthma are further addressed later in this review.

### 2.2. Mechanisms of Action of GCs

Due to their lipophilic nature, GCs rapidly diffuse across the cell membrane to the cytoplasm, where they interact with the GC receptor (GR) [31]. This interaction promotes the conformational change in GR and its subsequent separation from chaperone proteins, e.g., heat shock protein 90 (hsp90) and p23 [7]. The GR will then translocate to the nucleus, where it homodimerizes with another GR molecule and acts as a transcription factor capable of activating gene expression by binding to GC response elements (GREs). This process, referred to as transactivation, promotes the expression of anti-inflammatory genes such as IL-10, mitogen-activated kinase phosphatase-1 (MKP-1), lipocortin-1, and IKK (IκB) [31,32]. In addition, as a monomer, GR can physically interact with a variety of inflammatory transcription factors (TFs) such as nuclear factor-κB (NF-κB) and activator protein-1 (AP-1) and interferes with their ability to induce the expression of pro-inflammatory genes such as IL-1 and TNFα [6,7,31]. This process is referred to as transrepression, where the GR/TF complex binds to the promoter region of inflammatory genes and subsequently recruits histone deacetylase (HDAC)-2, thereby reversing histone acetylation and inhibiting transcription [33,34].

### 2.3. Effects of GCs on Immune and Airway Cells

In GC-sensitive patients with asthma, GCs significantly reduce airway inflammation by affecting both immune cells and airway structural cells’ responses. For instance, GCs reduce eosinophil survival by inhibiting the expression of granulocyte macrophage colony-stimulating factor (GM-CSF) [35]. Similarly, GCs inhibit the activation of lymphocytes and promote their apoptosis in vitro while reducing their numbers in the blood of patients with asthma when given orally [23]. Other immune cells affected by GC treatment include macrophages by inhibiting their cytokine and chemokine secretion [24], monocytes by reducing their peripheral blood numbers and the expression of IgE receptor [36], and dendritic cells by modulating their migration to local lymph nodes [37]. Moreover, GCs affect the expression of adhesion molecules and the secretion of chemokines in immune cells, thereby reducing their infiltration into peripheral tissues [38,39]. Interestingly, GCs also affect the function of airway structural cells, such as ASM cells. The effects of GCs in ASM cells were comprehensively reviewed in our recent publication [40]. Briefly, some pro-inflammatory genes that have the potential to regulate asthma pathogenesis have been reported to be inhibited by GCs in ASM cells. For instance, dexamethasone or fluticasone propionate (FP) suppressed TNF-induced production of various chemokines, including CXCL8 [41,42], CCL5 and IL-6 [43,44], CCL11 [45], and CXCL10 [46], and the expression of intercellular adhesion molecule 1 (ICAM-1) [47]. Moreover, responses induced by IL-1β, such as the expression/activity of matrix metalloproteinase 12 (MMP-12), the production of CXCL10 and GM-CSF, or the expression of ICAM-1, were also reported to be inhibited by dexamethasone [47,48,49,50] or FP [51]. In addition, GCs were shown to be effective in inhibiting the production of pro-inflammatory mediators, including IL-6 and CXCL8, induced by G protein-coupled receptor (GPCR) agonists such as bradykinin [52,53,54] or sphingosine-1 phosphate (S1P) [55]. Furthermore, ciclesonide, a GC that must be converted by desisobutyryl-ciclesonide by lung esterases to be clinically active, and FP were equally effective in inhibiting the induction of CCL2 in response to TNF stimulation [56,57]. Interestingly, GCs exert a differential suppressive effect on the expression of pro-inflammatory genes in ASM cells, and not all genes are repressed with equal potency/efficacy. For example, induction of some genes such as IL-6, CCL2, CCL5, or CXCL10 appears to be strongly inhibited by dexamethasone or FP (>80–90% inhibition at 10^−5^M), while other responses such as expression of ICAM-1, CXCL8, CCL11, or GM-CSF were found to be only partially repressed (50–60% inhibition at 10^−5^–10^−6^M). Surprisingly, other genes such as IL-33, CX3CL1, TARC, or CCL11 were found to be unaffected by either dexamethasone or FP [58,59,60,61]. Collectively, these studies suggest that GCs exert a strong anti-inflammatory action in ASM cells on a variety of inflammatory genes induced by pro-asthmatic stimuli, although the potency/efficacy appears to be highly gene- and stimuli-specific.

## 3. Glucocorticoid Insensitivity

### 3.1. Clinical Definition of GC Insensitivity in Asthma

As mentioned above, the sensitivity to GC treatment often decreases with asthma severity. Importantly, the lack of GC sensitivity is not limited to asthma and also manifests in other chronic inflammatory diseases, such as chronic obstructive pulmonary disease (COPD), rheumatoid arthritis, and inflammatory bowel disease [62]. To understand the mechanisms involved in GC insensitivity, it is important to consider the degree to which subjects are (in) sensitive to GC treatment. In several studies, patients with asthma are defined as GC-resistant if they show less than 10% improvement in their pre-bronchodilator morning forced expiratory volume during 1 s (FEV1) predicted response after a one-week course of 40 mg/day of oral prednisone, and as GC-sensitive when they show a higher improvement (≥12%) [63,64]. Others used slightly different standards, such as defining GC resistance as having less than 15% improvement in baseline pre-bronchodilator FEV1 after one month of inhaled corticosteroids [65]. A better understanding of the cellular and molecular mechanisms that drive a suboptimal response to GC treatment is key to improve the effectiveness of GC therapy in severe asthma.

### 3.2. Role of Airway Structural Cells in GC Insensitivity

Given the immunomodulatory role of ASM in asthma pathogenesis, studying the effects of GCs on ASM functions could generate critical findings needed to develop therapeutic strategies aimed at improving the efficacy of GC treatment in asthma. GR expression in ASM from patients with and without asthma has been established through in situ hybridization and immunohistochemistry studies [66]. Functional studies further showed that GC treatment induces GR nuclear translocation in cultured ASM cells derived from healthy subjects or from patients with asthma or emphysema [67]. In addition, activation of the GR by GCs through binding to GRE-DNA sequences in the promoters of GC-dependent genes has been demonstrated in ASM cells [67,68,69]. Current literature supports an effective role for GCs in suppressing ASM functions. For instance, the induction of several inflammatory genes, such as IL-6, RANTES [44], ICAM-1 [47], eotaxin [45], CD38 [68], GM-CSF [49], and cyclo-oxygenase-2 (COX-2) [70], is effectively suppressed in vitro by GCs in human ASM cells. The effects of GCs on inflammatory gene expression in ASM cells appear to be disease state-dependent. For instance, TNFα-induced CCL11, CXCL8, or IL-6 release was less susceptible to suppression by dexamethasone in ASM cells obtained from patients with severe asthma when compared to those obtained from healthy subjects [71,72]. Furthermore, while dexamethasone reduced the proliferation of ASM cells derived from healthy subjects, dexamethasone failed to inhibit the proliferation of ASM cells derived from patients with non-severe and severe asthma [71]. Interestingly, studies in endobronchial biopsies derived from patients with asthma reveal an increase in the expression of certain genes involved in asthma progression such as disintegrin and metalloprotease (ADAM) 33, ADAM8, eotaxin, and CCL19 in ASM layer [73,74,75]. Importantly, such an increase correlated with disease severity and with the lack of GC responsiveness. Altogether, these findings clearly demonstrate the existence of GC-insensitive pathways in ASM derived from patients with asthma whose activation may increase disease severity.

## 4. Common Pitfalls Associated with Studies Investigating GC Signaling

### 4.1. GC Resistance versus GC Insensitivity

While the terms “GC resistance” and “GC insensitivity” have been erroneously used interchangeably, these terms indicate different states of GC responsiveness. While “GC resistance” reflects a state of complete insensitivity to GC actions, “GC insensitivity” represents a spectrum of partial GC responsiveness [76].

### 4.2. Total GR Phosphorylation versus GR Site-Specific Phosphorylation

GR is subjected to several post-translational modifications. For example, GR phosphorylation is a critical step in the receptor activation as it affects the receptor’s ligand and DNA binding, its subcellular localization, its half-life, and, ultimately, its biological activity [77,78]. GR protein structure consists of an N-terminal domain (NTD) with a potent transactivation domain (AF-1), a DNA-binding domain (DBD), and a ligand-binding domain (LBD) with another transactivation domain (AF-2) [33,34]. In the AF-1 domain, the major functionally important phosphorylation sites are serine 203 (Ser203), Ser211, and Ser226 [25]. Evidence from cells lacking endogenous GR (e.g., U2OS/COS7 cells), transformed cells (e.g., rat hepatoma cells or leukemia cell lines), or yeast systems showed that GR activation is maximal when the relative phosphorylation of GR at Ser211 surpasses that of Ser226 [10,12,23,35]. These latter studies also showed that Ser211 increases, whereas Ser226 decreases, GR transcriptional activities.

The role of GR phosphorylation in GC insensitivity has long been controversial. Initially, total GR phosphorylation was associated with GC insensitivity, as earlier reports in PBMCs by Irusen and colleagues [79] and Li and colleagues [80] demonstrated that total GR phosphorylation reduced the activity of GR, thereby promoting GC insensitivity. Mechanistically, IL-2- and IL-4-induced p38 MAPK activation increased total GR phosphorylation and subsequently impaired GC effectiveness [79]. Furthermore, when PBMCs were stimulated with superantigen, cells became insensitive to the GC anti-proliferative effects. Interestingly, this effect was prevented by the inhibition of mitogen-activated protein kinase (MEK/ERK) activation [80].

The association between GR site-specific phosphorylation (rather than GR total phosphorylation) and GC responsiveness began to gain significant consideration when Miller and colleagues demonstrated that a GR mutation at Ser211 diminished GC ability to induce apoptosis in lymphoid cells [81]. Later, comprehensive studies conducted by Garabedian’s group showed that GR site-specific phosphorylation regulated the expression of GC target genes [82,83].

**Figure 1:**
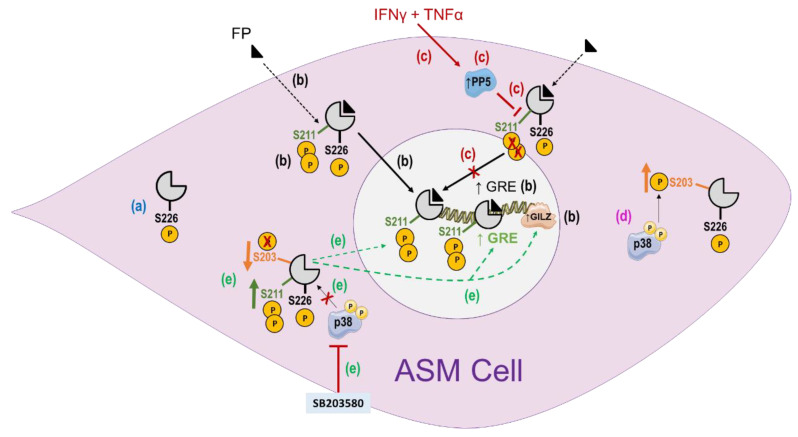
The role of GR site-specific phosphorylation in regulating GC response in airway smooth muscle (ASM) cells. (**a**) GR is constitutively phosphorylated at Ser226. (**b**) Phosphorylation at Ser211 is critical for its transcriptional activity. Glucocorticoids (GCs) such as fluticasone propionate (FP) increase GR phosphorylation at Ser211, its subsequent nuclear translocation, and its DNA binding to glucocorticoid responsive elements (GRE) present in the promoters of GC-dependent genes such as GILZ to induce their transcription. (**c**) Treatment with IFNγ/TNFα dramatically suppresses GC-induced phosphorylation at Ser211 but not at Ser226. PP5 mediates the IFNγ/TNFα suppressive effect on GR phosphorylation at Ser211. (**d**) Under resting conditions, p38 MAPK-dependent phosphorylation of unliganded GR at Ser203 preserves the inactive state of GR. (**e**) Pharmacological inhibition of p38 MAPK, using SB203580, reduces unliganded GR phosphorylation at Ser203 but increases its phosphorylation at Ser211, leading to its nuclear translocation and transactivation activities of GR, as evidenced by an increase in GRE reporter activity and GILZ expression.

The role of GR-site specific phosphorylation in regulating GC responsiveness in cells relevant to the pathogenesis of asthma, such as ASM cells, was first examined in our previous studies. For instance, we showed that GR was constitutively phosphorylated at Ser226, but not at Ser211. Importantly, cell stimulation with the pro-asthmatic cytokines TNFα and IFNγ dramatically reduced GC-induced GR phosphorylation at Ser211, thereby promoting GC resistance [84] (Figure 1). Additional work in human ASM cells revealed that the inhibition of p38 MAPK promotes GR nuclear translocation and GR-dependent induction of GC-target genes in the absence of any GCs/ligands. Such p38-dependent regulation of GR function was associated with GR phosphorylation at Ser203. Additional experiments showed that the inactive state of the GR under resting conditions is not only preserved by the absence of GCs but also relies on p38 MAPK-dependent phosphorylation of the “unliganded” GR at Ser203 [85] (Figure 1). Further studies in PBMCs obtained from patients with severe asthma showed an association of GC insensitivity with p38 or c-Jun N-terminal kinase (JNK)-dependent increases in GR site-specific phosphorylation at Ser226 [86,87,88,89]. Altogether, these studies highlight the functional importance of site-specific GR phosphorylation in regulating GC responsiveness in target cells/tissues.

### 4.3. Transactivation versus Transrepression Mechanisms

Traditionally, GCs were thought to exert their anti-inflammatory effects only through GR-mediated transrepression activities, whereas unwanted side effects were often attributed to GR-mediated transactivation activities. This concept has proven to be oversimplified, as the contribution of GR-mediated transactivation activities to the anti-inflammatory actions of GCs was underappreciated. However, several studies demonstrated the contribution of GC-inducible/target genes to the anti-inflammatory effects of GCs (Table 1).

For instance, in human ASM cells, GCs rapidly upregulate the mitogen-activated kinase phosphatase-1 (MKP-1), thus inhibiting TNFα-induced p38 MAPK phosphorylation and activities. Since TNFα stabilizes IL-6 mRNA transcript in a p38 MAPK-dependent fashion, GC-induced MKP-1 may contribute to the repression of IL-6 secretion in ASM cells [90]. This was clearly demonstrated by the ability of MKP-1 siRNA to prevent GC suppressive effects on TNFα-induced IL-6 secretion [90]. Similarly, other studies in ASM cells showed that dexamethasone inhibited IL-6 expression induced by the potent bioactive sphingolipid sphingosine 1-phosphate (S1P) without affecting CREB/CRE transrepression, IL-6 mRNA stability, or subcellular relocation of mitogen and stress-activated protein kinase 1 (MSK1). Rather, these effects were driven by rapid upregulation of MKP-1 by dexamethasone and subsequent inhibition of MAPK-induced activation of MSK1 and histone H3 phosphorylation [91]. Additional evidence supporting the contribution of GC-inducible/target genes to the anti-inflammatory effects of GCs comes from studies in airway epithelial cells. In these cells, the expression of glucocorticoid-induced leucine zipper (GILZ) is enhanced by dexamethasone. Interestingly, GILZ siRNA prevented the suppressive effects of dexamethasone on IL-1β-induced IL-8 expression, suggesting that GC-induced GILZ contributes to the repression of chemokine secretion in airway epithelial cells [93] (Table 1).

Recently, we identified a new GC-target gene, namely, insulin-like growth factor-binding protein 1 (IGFBP1), able to mediate the repressive effects of GCs in ASM cells [92]. We found that FP increased IGFBP1 mRNA and protein levels in these cells. The addition of exogenous/recombinant IGFBP-1 inhibited proliferation in cells derived from patients with asthma irrespective of growth factors used as stimuli. Further investigations of different signaling molecules involved in ASM growth, such as protein kinase B (PKB/AKT), mitogen-activated protein kinases (MAPKs), or focal adhesion kinase (FAK), showed that IGFBP-1 selectively decreased mitogen-induced p38 phosphorylation in these same cells. These findings clearly suggest that GC-dependent induction of IGFBP1 contributes, at least in part, to the anti-proliferative effects of GCs in ASM cells [92]. Interestingly, additional studies in A549 lung epithelial cells showed that cell treatment with recombinant IGFBP1 dramatically inhibited IFNγ actions by interfering with STAT1 phosphorylation and nuclear translocation [95], demonstrating, therefore, the contribution of GC-induced IGFBP1 to the anti-inflammatory effects of GC in airway epithelial cells [94].

## 5. Inflammatory Mediators Involved in Asthma Severity and Glucocorticoid Insensitivity

Glucocorticoid insensitivity (GCI) can be classified as primary or acquired GCI [96,97].

**Figure 2:**
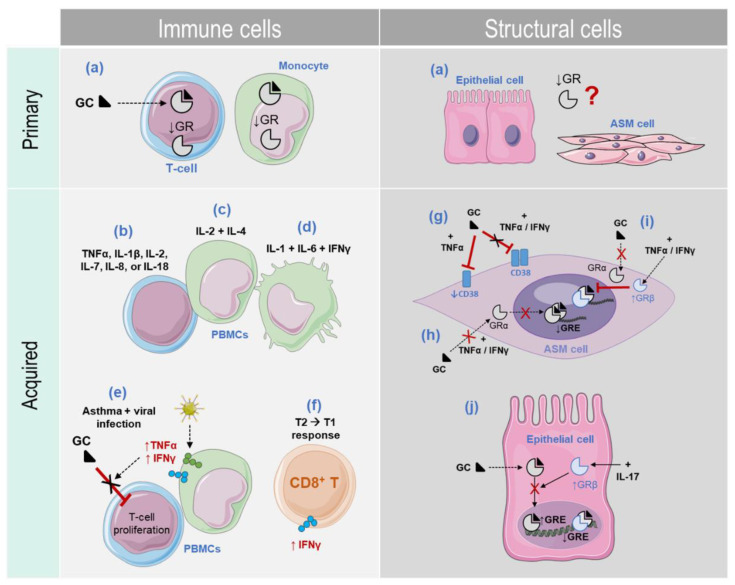
Glucocorticoid insensitivity (GCI) in asthma. GCI can be classified as primary or acquired. (**a**) Primary GCI is characterized by a low number of GR in immune and non-immune cells. (**b**–**f**) *Acquired GCI in immune cells*. In vitro studies performed in immune cells showed that acquired GCI can be promoted by mono- (**b**), bi- (**c**), and multi-stimulation (**d**) with inflammatory cytokines. (**e**) While PBMCs derived from patients with asthma during respiratory viral infection produce high levels of TNFα and IFNγ, T-cells obtained from the same patients are less responsive to GC treatment. (**f**) Peripheral blood lymphocytes (PBLs) obtained from patients with severe asthma show a predominant T1 cytokine response during exacerbation periods where CD8^+^ T-cells produce high levels of IFNγ. (**g**–**j**) *Acquired GCI in structural cells*. GCI also occurs in non-immune lung cells. (**g**) For instance, in ASM cells, GCs inhibit the expression of CD38 when cells were treated with TNFα alone, but not when cells were treated with the combination of TNFα/IFNγ. (**h**) TNFα/IFNγ co-treatment inhibits GC-mediated GRα-DNA binding activity and GRE-dependent gene transcription. (**i**) TNFα/IFNγ combination augments the GRβ levels, which inhibits GRα actions. (**j**) In lung epithelial cells, while FP increases GRE reporter activity, adding IL-17 reduces such an effect through the upregulation of GRβ expression.

Primary GCI, also called endogenous or intrinsic GCI, is irreversible and independent of inflammation. This form of GCI is rare and characterized by a mutation of the gene encoding the GR, thereby reducing its expression levels [96] (Figure 2).

Acquired GCI, however, does heavily depend on inflammation and represents the most frequent form of GCI [76]. Initially, acquired GCI was described to occur only in immune cells such as PBMCs and airway T-cells [98]. For instance, different in vitro studies showed that acquired GCI can be promoted by mono- (TNFα, IL-1β, IL-2, IL-7, IL-8, or IL-18), bi- (IL-2 and IL-4) [98], or multi- (IL-1, IL-6, and IFNγ) stimulation with inflammatory cytokines [99] (Figure 2). Amongst these cytokines, the involvement of TNFα and IFNγ in GCI in asthma received significant attention over the past decades. A pioneering study by Vianna and colleagues [100] showed that the anti-proliferative effects of dexamethasone in T-cells obtained from patients with asthma during respiratory infection, but not during the recovery phase, were significantly reduced. Such a reduction was associated with high expression levels of TNFα and IFNγ.

While acquired GCI has been described to occur only in immune cells [98], we and others have demonstrated that it can also occur in non-immune cells such as airway structural cells [72] (Figure 2). For instance, when we exposed ASM cells to TNFα and IFNγ, GC cellular responsiveness was significantly reduced [68]. When the expression of CD38 was examined in ASM cells, a molecule involved in AHR and airway inflammation in asthma, GC was unable to inhibit CD38 expression when cells were treated with the combination of TNFα and IFNγ [68]. Follow-up studies demonstrated that the combination of TNFα and IFNγ inhibited the GC-mediated GRα-DNA binding activity and GRE-dependent gene transcription to impair GC responsiveness. In addition, this cytokine combination markedly augmented GRβ levels, thereby altering GR functions and actions. Further mechanistic findings regarding these observations will be discussed below. Collectively, against the common belief that acquired GCI can occur only in immune cells, these findings clearly indicate that acquired GCI can also develop in non-immune cells, such as structural cells, highlighting, therefore, the novel and potential contribution of these cells to GCI seen in patients with severe asthma [68] (Figure 2).

Increased IFNγ levels were also reported in patients with chronic inflammatory airway diseases. For instance, the dominant cytokine profile in peripheral blood lymphocytes obtained from patients with severe asthma specifically during exacerbation periods was a T1 profile [101]. Moreover, high numbers of IFNγ-producing CD8^+^ T-cells were increased and correlated with asthma severity [102]. IFNγ levels are also significantly high in COPD patients [103]. For example, IFNγ serum levels were increased in patients with acute exacerbation of COPD as compared to patients with stable COPD [104]. Importantly, IFNγ production by airway lymphocytes obtained from COPD patients was insensitive to GC treatment [105]. Moreover, we also showed that IFNγ was able to reduce GC responsiveness in lung epithelial cells [68] by affecting the ability of GC to induce GRE reporter activity [106]. Collectively, these observations suggest that IFNγ, previously thought to be protective in asthma [107], could be pathogenic in a specific population of asthma (T2-low, chronic, severe) and COPD (with acute exacerbations) patients and can contribute to the poor GC responsiveness seen in these patients [101,102,108] (Figure 2).

Collectively, while IL-2 and IL-4 is the most common cytokine combination used to promote GCI in immune cells, these findings propose that IFNγ either alone or in combination with TNFα can promote GCI in non-immune cells such as ASM cells. Interestingly, since cytokines and the associated pathways interfere with GC responsiveness and promote GCI, it is legitimate to speculate that therapies targeting these inflammatory pathways such as biologics could restore GC responsiveness in patients with severe asthma. However, studies are still needed to further explore such therapeutic strategy in patients with different asthma phenotypes with various degrees of inflammation involvement such as granulocytic and paucigranulocytic asthma [109].

## 6. Common and Distinct Molecular Mechanisms Underlying the Impairment of Glucocorticoid Responsiveness in Immune Cells versus Airway Structural Cells

While most of the studies investigating the mechanisms underlying GCI in asthma have been conducted in immune cells, emerging findings from non-immune cells such as airway structural cells identified additional mechanisms. We highlight below some of the common and distinct mechanisms underlying the impairment of GC responsiveness in immune cells versus airway structural cells.

### 6.1. GRβ

As stated above, GRβ has been shown to act as a dominant negative receptor, with high levels seen in GC-insensitive patients [29,30]. GRβ is expressed in various immune cells, particularly in neutrophils [23]. In airway structural cells, several studies reported the functional expression of GRβ. For instance, in ASM cells, we demonstrated that GRβ overexpression resulted in the prevention of GRα-mediated trans-activation activities [68,110]. We also found that cell treatment with TNFα and IFNγ combination interfered with GRα/GRβ dynamics in favor of GRβ, thus reducing GC responsiveness [68]. Further evidence involving GRβ in GCI in airway structural cells stems from studies in epithelial cells. For example, studies in A549 cells showed that the ability of FP to increase GRE reporter activities, a reporter used to monitor GRα-mediated trans-activations activities, was completely suppressed by IL-17. Such suppression was markedly prevented when GRβ expression was reduced by siRNA [111].

GR isoforms are generated from the same pre-mRNA transcript through alternative splicing mechanisms, where factors controlling this process are of great importance. Serine/arginine-rich proteins (SRps) are known to be involved in both constitutive and alternative splicing processes. In neutrophils, for instance, cells highly insensitive to GC actions due to high GRβ levels, the selective reduction of SRp30c, but not other SRp subtypes, significantly decreased GRβ expression while restoring GC responsiveness [112]. Interestingly, we also examined whether the effects of IL-17 on GRβ expression in epithelial cells were due to an increase in SRps expression. We found that while IL-17 did not affect SRp30 expression, it specifically increased SRp40 expression, suggesting that, unlike in immune cells, SRp40 mediates GRβ upregulation in airway structural cells [113]. However, further studies are still needed to validate these observations.

#### GRβ and HDAC2 Crosstalk

The reduction in HDAC activity has been associated with GCI [114]. For instance, the expression of HDAC2 was dramatically reduced in patients with severe asthma and COPD. This effect was mainly due to increased oxidative stress processes and the activation of phosphoinositide 3-kinase (PI3K) δ. When antioxidants and Nrf2 activators were used to reduce oxidative stress and thereby PI3Kδ activity, the expression of HDAC2 was increased and the efficacy of GCs was improved in macrophages obtained from these patients [34], clearly indicating a role of HDAC2 in modulating GCI in patients with severe asthma and COPD. Strikingly, Li and colleagues demonstrated that the increased GRβ expression and the decreased HDAC2 expression in macrophages are not necessarily mutually exclusive and revealed that GRβ activation can interplay with HDAC2 to promote GCI [115]. The authors showed that the ability of GC to increase the expression of HDAC occurs through the binding of GRα to GRE sites in the HDCA2 promoter. When GRβ expression was increased in patients with severe asthma, it interfered with GRα-mediated transactivation activities and specifically with the ability of GRα to bind GRE sites in HDAC2 promoter, thereby decreasing HDAC2 expression [115].

Collectively, these findings clearly indicate that GRβ is also expressed in non-immune cells such as airway structural cells, where it interferes with GC cellular responsiveness, suggesting the potential contribution of GRβ to the overall GCI seen in patients with severe asthma. Of note, the role of GRβ in regulating GR functions during inflammatory conditions was comprehensively addressed in our recent review [116].

### 6.2. Aberrant Activation of Inflammatory Transcription Factors

Aberrant activation of certain inflammatory transcription factors has been implied in the development of GCI. The most studied inflammatory transcriptional factors known to reduce GC actions are NF-κB and AP-1. For instance, mutual antagonism between NF-κB and GR is well-documented, where NF-κB physically interacts with GR and suppresses its activity, interfering with GC responsiveness [117]. Moreover, crosstalk between AP-1 and GR has also been established in PBMCs obtained from GC-resistant patients with asthma, where the augmented levels of AP-1 prevented GR-DNA binding, thus providing a potential molecular mechanism driving GCI in immune cells [118].

Our group identified an additional transcription factor whose activation interferes with GC signaling in airway structural cells. Indeed, we showed that interferon regulatory factor-1 (IRF-1), a transcription factor induced by several pro-asthmatic cytokines, promotes GCI in ASM cells through the suppression of GRα-mediated activities and the inhibition of GRE-dependent gene transcription [119]. Further mechanistic studies revealed that, unlike NF-κB and AP-1, competition for a common co-activator such GR interacting protein-1 (GRIP-1) appears to be the main mechanism by which IRF-1 interferes with GR signaling [120].

Collectively, apart from NF-κB and AP-1, findings from airway structural cells revealed the involvement of novel transcription factors, i.e., IRF-1, whose aberrant activation interferes with GR function and GC cellular responsiveness. However, further in vivo studies are still needed to validate the role of IRF-1 in promoting GCI in patients with severe asthma.

### 6.3. Serine/Threonine Protein Phosphatases and Kinases

As stated above, GR phosphorylation is a critical step in the receptor activation. Because GR phosphorylation is a reversible mechanism, it is legitimate to postulate that GCI seen in patients with severe asthma may be due to an impairment of GR phosphorylation. Moreover, GR phosphorylation is highly cell-, tissue-, species-, and promoter-specific, which explains, at least partially, the controversy surrounding its role in overall cellular GC responsiveness. Part of this controversy may also be explained by the variety of serine/threonine protein phosphatases (PPs) and kinases that modulate GR phosphorylation.

#### 6.3.1. Serine/Threonine Phosphatases (PPs)

Several studies reported the differential involvement of PPs in the regulation of GR phosphorylation in both immune and non-immune cells. For instance, in immune cells such as PBMCs, PP2A has been implicated in the positive regulation of GR functions and the enhanced GC cellular responsiveness. Mechanistic studies in U937 cells, a macrophage cell line, showed that PP2A dephosphorylates the GR at Ser226, a residue known to inhibit GRα functions. Inhibition of PP2A, either by pharmacological inhibitor or by siRNA, increased GR-Ser226 phosphorylation, which in turn inhibited GR nuclear translocation, reducing GC sensitivity [89]. Interestingly, in PBMCs derived from GC-resistant subjects, the expression and the activity of PP2A were reduced [89]. In accordance, exposure of U937 cells to IL-2/IL-4 enhanced GR phosphorylation at Ser226 and reduced GC sensitivity. Interestingly, the treatment of these latter cells with long-acting β2-adrenoceptor agonist (LABA) formoterol increased the expression and activation levels of PP2A while restoring GC responsiveness [121]. These findings could explain, at least in part, how LABAs enhance the anti-inflammatory effects of GCs, and further highlight their benefits as add-on therapy for the treatment of diseases, including severe asthma, where GC insensitivity is an issue [121].

Surprisingly, when the functional involvement of PP2A in the regulation of GR phosphorylation in airway structural cells was examined, no effect on GC cellular responsiveness was observed [84]. Strikingly, in airway structural cells, another PP was involved in the regulation of GR-site phosphorylation. When ASM cells were treated with TNFα and IFNγ combination, the expression of PP5, but not PP2A or PP1, was increased, while GR phosphorylation at Ser211 and GC sensitivity were reduced [84]. Mechanistically, PP5 dephosphorylates GR at Ser211, a residue known to activate GRα functions, as evidenced by PP5 siRNA studies [84]. Additional in vivo studies showed an increase in PP5 expression in ASM bundles obtained from endobronchial biopsies of patients with severe asthma [122]. These data clearly indicate that in airway structural cells, PP5-mediated impairment of GR-Ser211 phosphorylation could contribute to GCI induced by pro-asthmatic cytokines [84].

Together, these findings highlight the cell-specific nature of PPs in regulating GR site-specific phosphorylation and function in immune versus non-immune/airway structural cells.

#### 6.3.2. Kinases

Various studies described the role of different kinases in the modulation of GR function and actions. In immune cells, most MAPKs have been shown to phosphorylate GR at Ser226 while reducing GR nuclear translocation and GC cellular responsiveness [34]. For example, while IL-2 and IL-4 promote GCI in PBMCs cells, the addition of p38 MAPK inhibitors restored GC cellular responsiveness, indicating the negative role of p38 in the modulation of GR function and GC responsiveness. Currently available inhibitors of p38-MAPK selectively inhibit α and β isoforms but not the γ isoform. However, this isoform has emerged to play a major role in GCI, and its expression was upregulated in PBMCs obtained from patients with severe asthma [87]. Interestingly, reducing the expression and activity of p38-MAPK-γ isoform by siRNA restored GC responsiveness in IL-2/IL-4-treated PBMCs [87]. Additional studies aimed at developing and examining p38-specific inhibitors are still needed. Interestingly, the involvement of JNK in regulating GR phosphorylation in other immune cells has also been demonstrated [123].

In contrast to immune cells, p38 MAPK negatively regulates GC responsiveness in ASM cells via direct GR phosphorylation of Ser203 but not Ser226 residues. Indeed, we previously showed that while the inhibition of p38-MAPK using SB203580 in ASM cells increased GR phosphorylation at Ser211, it decreased GR phosphorylation at Ser203 and had no effect on GR phosphorylation at Ser226 [85]. Additional mechanistic studies using GR-Ser203A mutant, MAPK kinase 3 expression vector (a specific kinase that directly activates p38 MAPK), and p38 active kinase demonstrated that the inactive state of the GR under resting conditions is not only preserved by the absence of GR ligands but also relies on p38 MAPK-dependent phosphorylation of the “unliganded” GR at Ser203. Treatment of ASM cells with GC reduced basal p38 MAPK expression as well as basal GR-Ser203, thereby enhancing GR-Ser211 phosphorylation and GR-mediated transactivation activities [85].

Together, these studies clearly indicate that inflammatory kinases differentially regulate GR phosphorylation and function in immune cells versus non-immune cells, where special attention needs to be given when designing kinase inhibitors to mitigate GCI in patients with severe asthma.

## 7. Concluding Remarks

Traditional views describe GCI as strictly localized in immune cells. We and others demonstrated that GCI encompasses immune cells to involve airway structural cells such as ASM cells. Importantly, both common and distinct mechanisms underlying the development of GCI have been revealed in immune versus non-immune cells. For instance, while IL-2 and IL-4 is the most common cytokine combination used to promote GCI in immune cells, IFNγ either alone or in combination with TNFα can promote GCI in non-immune cells, such as airway structural cells, since these latter cells do not express IL-2 receptor. Furthermore, GRβ has also been shown to be expressed in airway structural cells, where it interferes with GC cellular responsiveness. Furthermore, apart from NF-κB and AP-1, findings from airway structural cells revealed the involvement of novel transcription factors, i.e., IRF-1, whose aberrant activation interferes with GR function and GC cellular responsiveness. Strikingly, inflammatory kinases (p38 MAPK) and phosphatases (e.g., PP5 and PP2A) differentially regulate GR “site-specific phosphorylation” and function in immune cells versus non-immune cells. Collectively, these findings suggest the potential contribution of airway structural cells to the overall GCI seen in patients with severe asthma. These findings also suggest that special attention needs to be given to airway structural cells when designing future therapeutics aimed to restore GC responsiveness in GC-insensitive patients.

## Figures and Tables

**Table 1:** Examples of GC effects mediated by transactivation mechanisms in airway structural cells.

Cell Type	Mediators	Effect of GCs	Underlying Mechanism	Reference
**ASM Cells**	TNF-α	↓ IL-6 secretion	↑ MKP-1 ↓ p38 MAPK phosphorylation	[90]
	S1P	↓ IL-6 protein secretion and mRNA expression	↑ MKP-1 ↓ MAPK-induced activation of MSK1 ↓ Histone H3 phosphorylation	[91]
	PDGF, EGF	↓ Cell proliferation	↑ IGFBP1 ↓ p38 MAPK phosphorylation	[92]
**Airway** **Epithelial** **Cells**	IL-1β	↓ IL-8 mRNA ↓ NF-κB activation	↑ GILZ	[93]
	IFNγ	↓ STAT1 phosphorylation and nuclear translocation	↑ IGFBP1	[94,95]

## Data Availability

Not applicable.

## Abbreviations

ADAM	Disintegrin and metalloprotease
AF	Activation function
AHR	Airway hyperresponsiveness
AP-1	Activator protein-1
ASM	Airway smooth muscle
CCL11	Chemokine (C-C motif) ligand 11
CCL19	Chemokine (C-C motif) ligand 19
CD	Cluster of differentiation
COPD	Chronic obstructive pulmonary disease
COX-2	Cyclo-oxygenase-2
CREB/CRE	cAMP response element-binding protein/cAMP response elements
CTGF	Connective tissue growth factor
CXCL8	Chemokine (C-X-C motif) ligand 8
DBD	DNA-binding domain
FAK	Focal adhesion kinase
FEV1	Forced expiratory volume during 1 s
FP	Fluticasone propionate
GCI	Glucocorticoid insensitivity
GCs	Glucocorticoids
GILZ	Glucocorticoid-induced leucine zipper
GM-CSF GPCR	Macrophage-colony-stimulating factor G protein-coupled receptor
GR	Glucocorticoid receptor
GREs	Glucocorticoid response elements
GRIP-1	GR-interacting protein-1
HDAC	Histone deacetylase
hGR	Human glucocorticoid receptor
hsp90	Heat shock protein 90
ICAM	Intercellular adhesion molecules
IFNγ	Interferon gamma
IgE	Immunoglobulin E
IGF	Insulin-like growth factor-1
IGFBP1	Insulin-like growth factor-binding protein 1
IKK	IκB kinase
IL	Interleukin
IRF-1	Interferon regulatory factor 1
JNK	c-Jun N-terminal kinase
LABA	Long-acting β2-adrenoceptor agonist
LBD	Ligand-binding domain
MAPK	Mitogen-activated protein kinases
MEK/ERK	Mitogen-activated protein kinase
MKP-1 MMP-12	Mitogen-activated kinase phosphatase-1 Matrix metalloproteinase 12
MSK1	Mitogen and stress-activated protein kinase 1
NF-κB	Nuclear factor-κB
Nr3cl	Nuclear receptor 3, group C, member 1
Nrf2	Nuclear factor erythroid 2–related factor 2
NTD	N-terminal domain
PBMCs	Peripheral blood mononuclear cells
PDGF-BB	Platelet-derived growth factor
PI3K	Phosphoinositide 3-kinase
PKB/AKT	Protein kinase B
PPs	Protein phosphatases
RANTES	Regulated on activation, normal T cell expressed and secreted
S1P	Sphingosine 1-phosphate
Ser	Serine
siRNA	Small interfering RNA
SRps	Serine/arginine rich proteins
STAT1	Signal transducer and activator of transcription 1
TFs	Transcription factors
TGFβ	Transforming growth factor-β
TNFα	Tumor necrosis factor alpha
UTR	Untranslated regions
VEGF	Vascular endothelial growth factor
